# The impact of the COVID-19 pandemic on stress and other psychological factors in pregnant women giving birth during the first wave of the pandemic

**DOI:** 10.1186/s12978-022-01493-9

**Published:** 2022-09-05

**Authors:** Theresa Hübner, Tanja Wolfgang, Ann-Catrin Theis, Magdalena Steber, Lea Wiedenmann, Achim Wöckel, Joachim Diessner, Grit Hein, Marthe Gründahl, Ulrike Kämmerer, Sarah Kittel-Schneider, Catharina Bartmann

**Affiliations:** 1 Department of Obstetrics and Gynaecology, University Hospital of Würzburg, Josef-Schneider-Str. 4, 97080 Würzburg, Germany; 2 Department of Psychiatry, Psychosomatic Medicine and Psychotherapy, University Hospital, Margarete-Höppel-Platz 1, 97080 Würzburg, Germany

**Keywords:** COVID-19 pandemic, Concern, Depression, Anxiety, Maternal bonding, Self-efficacy

## Abstract

**Background:**

The onset of mental illness such as depression and anxiety disorders in pregnancy and postpartum period is common. The coronavirus induced disease 2019 (COVID-19) pandemic and the resulting public policy responses represent an exceptional situation worldwide and there are hints for adverse psychosocial impact, hence, the study of psychological effects of the pandemic in women during hospitalization for delivery and in the postpartum period is highly relevant.

**Methods:**

Patients who gave birth during the first wave of the COVID-19 pandemic in Germany (March to June 2020) at the Department of Obstetrics and Gynecology, University of Würzburg, Germany, were recruited at hospital admission for delivery. Biosamples were collected for analysis of SARS-CoV-2 infection and various stress hormones and interleukin-6 (IL-6). In addition to sociodemographic and medical obstetric data, survey questionnaires in relation to concerns about and fear of COVID-19, depression, stress, anxiety, loneliness, maternal self-efficacy and the mother–child bonding were administered at T1 (delivery stay) and T2 (3–6 months postpartum).

**Results:**

In total, all 94 recruited patients had a moderate concern of severe acute respiratory syndrome coronavirus 2 (SARS-CoV-2) at T1 with a significant rise at T2. This concern correlated with low to low-medium general psychosocial stress levels and stress symptoms, and the women showed a significant increase of active coping from T1 to T2. Anxiety levels were low and the Edinburgh Postnatal Depression Scale showed a medium score of 5 with a significant (T1), but only week correlation with the concerns about SARS-CoV-2. In contrast to the overall good maternal bonding without correlation to SARS-CoV-2 concern, the maternal self-efficiency correlated negatively with the obstetric impairment caused by the COVID-19 pandemic.

**Conclusion:**

Obstetric patients` concerns regarding SARS-CoV-2 and the accompanying pandemic increased during the course of the pandemic correlating positively with stress and depression. Of note is the increase in active coping over time and the overall good mother–child-bonding. Maternal self-efficacy was affected in part by the restrictions of the pandemic.

*Clinical trial registration* DRKS00022506

## Plain Language Summary

The global pandemic of COVID-19 (coronavirus induced disease 2019) is challenging our society in many ways. Especially pregnant women are facing extraordinary conditions and worries, like uncertain risks for mother and fetus in case of infection, restricted prenatal classes or restricted visitor regulations in hospitals. Particularly it is known that pregnancy and the postnatal period are presenting a more psychologically vulnerable time in a woman’s life. Developing the GeZeCO study, we aimed to focus on the pandemic’s effects on mental health of pregnant women during this time. Women giving birth in the department of obstetrics of the University Hospital Würzburg were asked to participate in the study. In total, 94 women completed several questionnaires concerning their mental health postpartum and again after 3 to 6 months. Further, we took blood samples of the women during the delivery stay and registered sociodemographic and obstetric data. Our results showed, that the women’s concern relating to COVID-19, as well as the level of depression and anxiety raised during the pandemic. In addition, the self-efficacy of the mothers was affected by the restriction measures. Despite this, the women had at large a good mother–child-bonding and their competence of active coping increased during time. In summary, we did find that the mental health of obstetric patients is impaired by the COVID-19 pandemic. This points out the importance of not only attending to physical health but also taking care of psychological stress and mental health problems of obstetric patients during this exceptional time.

## Introduction

In March 2020 the coronavirus induced disease 2019 (COVID-19) was declared a worldwide pandemic by the World Health Organization (WHO) [1] and confronts global society with new and unexpected challenges. The effects of this pandemic, which arrived in Germany in March 2020, do not only refer to the infection with severe acute respiratory syndrome coronavirus 2 (SARS-CoV-2) and its possible short and long-term consequences, but also death from COVID-19. Infection control measures including lockdown regulations affect the everyday life of each individual in various ways. In addition to wearing face masks and general hygiene requirements, this includes social contact restrictions, restriction of the range of movement, short-time work and/or the closure of workplaces, increased work-from-home arrangements as far as possible, restriction or closure of schools and kindergartens, restriction of shopping opportunities as well as quarantine measures in case of COVID-19 disease [2]. These government responses varied from country to country and showed an extraordinary range in the context of different health systems, political systems, economic interests, and attitudes regarding human rights [3–5]. In the medical field, in addition to strict hygiene measures at the beginning of the pandemic, examinations, treatments and operations that were not absolutely urgent were postponed in order to keep the highest possible number of intensive care beds available [6]. There was also a ban or at least restriction on hospital visitors.

Such situations due to an infectious pathogenic coronavirus strain were known to a more moderate extent from the severe acute respiratory syndrome (SARS) pandemic from 2002 until 2003 and the Middle East respiratory syndrome (MERS) epidemic of 2015 [7]. Lessons of these past pandemics showed that in the absence of vaccines or proven effective therapies prevention is the key to disrupt the chain of infections [8].

It is known that pregnancy and the postpartum time present one of the most mentally vulnerable periods in a woman´s life with a higher risk for the onset of depression and anxiety [9, 10]. In addition, maternal mental illness in the postpartum period can negatively affect the development of the child and therefore needs to be diagnosed and treated urgently and sufficiently [11]. Numerous investigations showed an impairment of mental health in the general population due to the COVID-19 pandemic and its restrictions [12–15]. Thus, the cohort of women pregnant during the pandemic seems to be a group of particular interest and concern regarding the possible effects of pandemic restrictions on mental health [16]. Based on experiences from disasters like earthquakes, hurricanes or terrorist attacks the exposure to disaster and associated stress can lead to an impairment of mental health of pregnant women and can have an impact on the pregnancy outcome [17]. Analogies to a global pandemic are to be expected.

At the beginning of the COVID-19 pandemic, no prior knowledge was available as to whether a maternal SARS-CoV-2 infection could be transmitted via the placenta to the fetus and what effects COVID-19 would have on the fetus and the course of pregnancy. Whereas Middle East respiratory syndrome coronavirus (MERS-CoV) and severe acute respiratory syndrome coronavirus (SARS-CoV) were found to be associated with adverse pregnancy outcomes as miscarriage or fetal death, preterm birth and a higher risk for severe maternal illness [18, 19] the current data published so far fails to prove any clear evidence for vertical transmission of SARS-CoV-2 to the fetus or newborn while the findings concerning a higher maternal risk for severe infection remain ambiguous [18, 20, 21]. Nevertheless, a Canadian study showed several changes in perinatal outcomes during the pandemic, for example a higher rate of obstetric intervention in early pregnancy [22]. Further, meta-analyses supported a higher risk of severe course of COVID-19 pregnancy and adverse pregnancy outcomes [23, 24]. An increasing prevalence for mental health problems like anxiety and depression of mothers and pregnant women was reported in previous studies [21, 25].

During the lockdown measures, there were also strict visitor regulations in German obstetric departments, hospital wards and delivery rooms. A higher stress level and prevalence of anxiety for pregnant women were shown at the onset of the pandemic and restrictive measures in previous studies [26–29].

Worldwide research at the beginning of the pandemic focused on SARS-CoV-2, possible therapeutic targets and preventive measures, as well as the COVID-19 pandemic and its various potential impacts on health care, mental health and economic effects [30–34]. Pregnant women were particularly observed regarding vertical transmission, fetal infection and pregnancy outcome during the first wave of the pandemic, while their mental health was less of priority [35]. As previous results show a general increase of mental health problems, women, especially in perinatal situations, seem to have a higher risk for impairment of mental health [36–38] or loneliness and isolation [39–41]. For this reason, the present study aimed to fill this gap in current knowledge.

The aim of the GeZeCO study was to detect the psychological effects of the COVID-19 pandemic in patients during their delivery stay and in the postpartum period in Germany. Therefore, we focused entirely on the effects of the pandemic on depression, stress and anxiety during course of time and as first examined the maternal self-efficacy and the mother–child bonding.

## Methods

### Study population

**Fig. 1:**
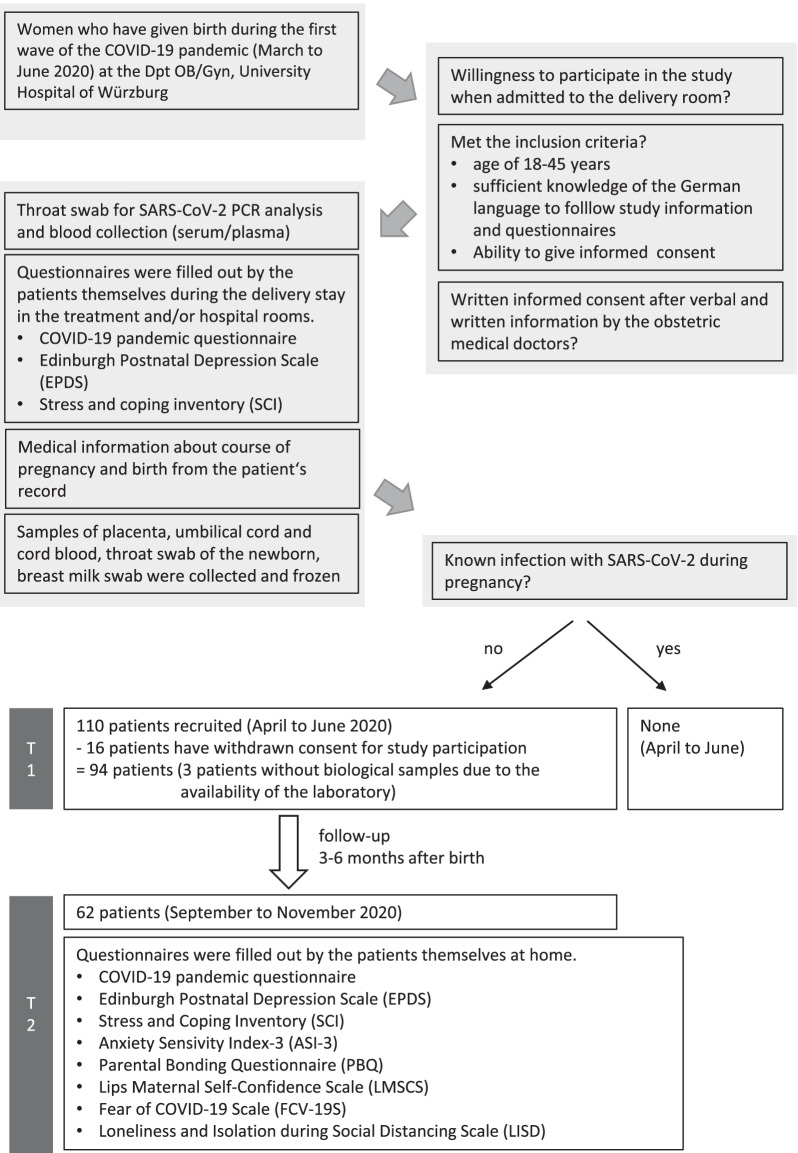
Entire study procedure

All woman admitted to our hospital, the Department of Obstetrics and Gynecology of the University Hospital of Würzburg, to give birth from April to June 2020 were asked upon the initial routine medical clarification interview at entry to the delivery room if they were willing to participate in the study, if they fit the inclusion criteria especial due to language aspects (in order to follow the questions). Only in the case of a large number of simultaneous births or emergencies that required all medical staff capacity, or if the laboratory was not available, no recruiting was performed. The obstetrician doctors were supported by a medical student (PhD) and a psychological student (Master's thesis) in clinical data collection and by the midwifes in biological sample collection. Details of the entire study procedure, the inclusion criteria and an overview of the questionnaires and the bio samples are shown in Fig. 1. The study was approved by the ethics committee of the University of Würzburg (No. 70/20 Amendment). After receiving verbal and written information, the patients agreed to participate in the study with written informed consent. The study adhered to the Declaration of Helsinki, version 2013.

### Laboratory analysis

Biosamples were collected for analysis of SARS-CoV-2 infection and various stress hormones and interleukin-6 (IL-6). Throat swabs were examined for the presence of SARS-CoV-2 at the Institute of Virology at the University of Würzburg or in the research lab of the Department of Gynecology using reverse transcriptase quantitative polymerase chain reaction (RT-qPCR) or reverse transcriptase polymerase chain reaction (RT-PCR) respectively. Enzyme-linked immunosorbent assays (ELISA) were performed with frozen blood serum samples according to manufacturer’s instructions in the research lab of the Department of Gynecology to test the immunoglobulins G and M of SARS-CoV-2 (EL-2006-9601 G, EL-2606-9601-2M, Euroimmun, Lübeck, Germany), cortisol (ARG81162, arigo biolab), adrenaline (E-EL-0045, Elbscience), norepinephrine (E-EL-0047, Elbscience), dopamine (E-EL-0046, Elbscience) and IL-6 (DY206-05, R&D).

### COVID-19 pandemic questionnaire (CPQ) and Fear of COVID-19 Scale (FCV-19S)

The first part of the self-designed COVID-19 pandemic questionnaire asked for specific symptoms of the diseases during pregnancy in an open-ended question and then for following symptoms; fever (temperature higher than 38.5 °C), cough, shortness of breath, muscle and joint pain, sore throat, headache, nausea/vomiting, nasal congestion, diarrhea, taste and/or smell problems and pneumonia. Finally, a possible contact with a SARS-CoV-2 positive person, a historical positive throat swab for SARS-CoV-2 and/or a COVID-19 disease were queried. The questions of the second part are shown in Table 1 and are divided into the concern scale (questions 1–4), the concern over time scale (questions 5–8) and the impairment scale (questions 9–11). The results of the scales were calculated as the sum of the appropriate questions. A Likert scale [42] was applied for questions 1–8 (1 = No, never; 2 = I have thought about it, but was not concerned; 3 = I am a little concerned; 4 = I am often concerned; 5 = I am concerned about it all the time) and questions 9–11 (1 = not at all; 2 = a little bit, 3 = moderately; 4 = quite a lot; 5 = a lot). The Fear of COVID-19 scale (FCV-19S) was published by Ahorsu and colleagues and the sum score of all questions was used to measure the fear of COVID-19 [43] and validated in German [44] and translated into at least 16 languages [45, 46].

**Table 1:** Questions and scales of the COVID-19 pandemic questionnaire and the results answered by 94 obstetric patients. Median and interquartile range of the following ordinal scale (1–5) is presented:

T1		Median	Interquartile range
Concern scale	1. Are/were you concerned about infecting yourself with the novel Coronavirus?	2.0	2.0–3.0
	2. Are/were you concerned that your unborn baby might get infected during pregnancy?	2.0	2.0–3.0
	3. Are/were you concerned that your newborn baby might be infected with the novel Coronavirus by you?	2.0	1.0–3.0
	4. Are/were you concerned that your newborn baby might be infected with the novel Coronavirus by others?	3.0	2.0–3.0
Concern over time scale	5. Are/were you concerned about being infected when the first European patient was reported?	2.0	1.0–2.0
	6. Were you concerned about being infected when the first European patient died?	2.0	1.0–2.0
	7. Were you concerned about getting infected when the number of infected people increased?	3.0	2.0–3.0
	8. Were you concerned about being infected when the exit restrictions (lockdown) went into effect?	2.0	2.0–3.0
Impairment scale	9. How badly is your quality of life affected by the COVID-19 pandemic?	3.0	2.0–4.0
	10. How severely is the course of pregnancy affected by the COVID-19 Pandemic?	3.0	2.0–4.0
	11. How badly is the birth of your baby affected by the COVID-19 pandemic?	2.0	2.0–4.0

For questions 1–8: 1 = no, never; 2 = I have thought about it, but was not concered; 3 = I am a little concerned; 4 = I am often concerned; 5 = I am concerned about it all the time

For questions 9–11: 1 = not at all; 2 = a little bit, 3 = moderately; 4 = quite a lot; 5 = a lot

### Stress and coping inventory (SCI)

The stress and coping inventory (SCI) is a German-language stress questionnaire with 54 items [47]. The first 21 items of the SCI are divided into three subscales consisting of seven items each: “stress caused by insecurity”, “stress caused by being overwhelmed” and “stress caused by loss”. Here, a seven-point Likert scale from “not burdened” to “very heavily burdened” is used. Together, the three subscales assess the total stress level. Here, a seven-point Likert scale from “not burdened” to “very heavily burdened” is used. The following 13 items measure physical stress symptoms on a four-point Likert scale (“does not apply at all”, “applies a bit”, “moderately applies” and “applies completely”). The same Likert scale is applied to the last 20 items evaluating coping strategies. The coping items can be divided into “positive coping”, “active coping”, “coping by support”, “coping by believing in God or powers that be” and “coping by drinking alcohol and/or smoking”, each with four items. For the evaluation of SCI scales, the sum of all item points of each scale was formed following the instructions of the evaluation manual [48].

### Depression and anxiety (EPDS and ASI-3)

The Edinburgh Postnatal Depression Scale (EPDS) was developed in 1987 as a screening instrument for postnatal depression [49] and translated and adapted to German [50–52]. The total score is the sum of all ten items with a four-point Likert scale (from 0 to 3). An EPDS value of 10 or higher has a middle to high probability for depression [53].

To detect anxiety we used the Anxiety Sensitivity Index-3 (ASI-3) that was developed by Taylor in 2007 [54] and translated as well as adapted to German by Kemper [55]. The response of the 18 items with a five-point Likert scale were summed up in a total score.

### Maternal self-confidence (LMSCS) and parental bonding (PBQ)

The Lips maternal self-confidence scale (LMSCS) was developed by Bloom and Lips in 1993 and translated to German [56]. It contains 25 questions with a six point Likert scale. The total score is the sum of each item.

The Parental Bonding Questionnaire (PBQ) is a self-reported questionnaire with 25 items. The answers of its six-point Likert scale have been added up to a total score [57, 58].

### The Loneliness and Isolation during Social Distancing Scale (LISD Scale)

The Loneliness and Isolation during Social Distancing (LISD) Scale questionnaire was developed and validated by Gründahl and colleagues on the occasion of the COVID-19 pandemic [59]. This questionnaire assesses loneliness and isolation in the context of social distancing on a state and trait level. The first part with 12 items concerns a person’s current experience and feelings (state) and is divided into two subscales (“lonely and isolated” and “connected and supported”). The second part of the LISD scale consists of 13 items and assesses a person’s experience and feelings in general (trait). It is divided into three subscales (“loneliness and isolation”, “sociability and sense of belonging” and “social support and closeness”). For each item, a five point Likert scale is used (ranging from “strongly agree” to “strongly disagree”).

### Statistical analysis

The sample size was calculated by a professional statistician. The software SPSS Statistics 26 (IBM) was used to perform statistical analyses and create tables. For normal distributions, Shapiro–Wilk tests were performed. Data are presented accordingly as mean (± standard deviation [SD]) or median (interquartile percentile) values. Wilcoxon test and Mann–Whitney-U-test were used to detect significant differences in non-parametric data. P-values ≤ 0.05 were rated as statistically significant. McDonald’s Omega was determined for the score reliability and Spearman's rho test for inter-scale correlation.

## Results

### Study population

A total of 94 patients were enrolled in the study from April to June 2020 (T1). Basic characteristics and obstetric information of the study population are shown in Table 2. 62 patients (66.0%) participated in the follow-up at 3 to 6 months (T2) after birth (September to November 2020). We collected biospecimens from 91 patients; 3 specimens could not be determined because of unavailability of the laboratory. From all patients we had the data from the questionnaires. Rarely, individual questions were not answered and were noted as missing. The number (n) was 92 ± 2 at T1 and 61 $\pm$ 1 at T2 in the analysis of all questionnaires.

**Table 2:** Basic characteristics and obstetric information of the study population

	Mean	SD	Number	In percent
Age in years	32.48	4.31		
Groups by age				
Age < 35 years			62	66.0%
Age ≥ 35 years			32	34.0%
BMI before pegnancy	23.95	4.34		
Groups by BMI				
BMI < 25			62	66.7%
BMI ≥ 25			31	33.3%
Education				
Non-academics^1^			59	62.8%
Academics^2^			35	37.2%
Previous mental illness				
No			87	92.5%
Yes			7	7.5%
Number of pregnancies				
1			36	38.3%
2			33	35.1%
3			12	12.8%
4			8	8.5%
5 and more			5	5.4%
Number of births				
1			49	52.1%
2			34	36.2%
3			9	9.6%
4 and more			2	2.2%
Number of vaginal births				
0			24	25.5%
1			42	44.7%
2			20	21.3%
3			6	6.4%
4 and more			2	2.2%
Number of Caesarian sections				
0			62	66.0%
1			26	27.7%
2			3	3.2%
3			3	3.2%
Number of miscarriages				
0			70	74.5%
1			15	16.0%
2			6	6.4%
3 and more			3	3.2%
Number of artificial abortions				
0			91	96.8%
1			3	3.2%
Mode of birth delivery				
Vaginal birth			62	66.0%
Vacuum extraction			3	3.2%
Scheduled Caeserian section			20	21.3%
Unplanned/emergency Caeserian section			9	9.6%
Date of birth				
Premature birth (before 37 weeks of pregnancy)			6	5.4%
Birth at term			78	94.6%
Multiples				
Singular			87	92.6%
Twin			7	7.5%
High-risk pregnancy^3^				
No			45	47.9%
Yes			49	52.1%
Complication during birth^4^				
No			65	69.1%
Yes			29	30.9%

SD = standard deviation

^1^Non-academics were women without an university degree

^2^Academics were university graduates

^3^High-risk pregnancy is defined according to risk catalogue B of the German maternity card which includes any complication in pregnancy

^4^Complication during birth were defined by changing mode of delivery and/or high blood loss (vaginal birth > 500 ml, caesarian section > 1000 ml) and/or fetal acidosis (pHarterial < 7.10, base excess < − 10 mmol/l)

### Infection status

None of the patients had knowingly suffered from COVID-19 and none were tested positive for SARS-CoV-2 by RT-qPCR or RT-PCR. 16 (17.0%) patients reported no respiratory infection during pregnancy, but 70 patients (74.5%) specified one or more of the following symptoms: 6 (6.4%) fever, 23 (24.5%) cough, 26 (27.7%) shortness of breath, 21 (22.3%) muscle and joint aches, 28 (29.8%) headache, 28 (29.8%) throat sore, 27 (28.7%) nausea and vomiting, 18 (19.1%) diarrhea, 47 (50.0%) congested nose, 8 (8.5%) new loss of taste and smell, none (0%) pneumonia. No patients had positive IgM antibodies against SARS-CoV-2, but two patients had positive IgG antibodies. The first of those two patients reported symptoms like shortness of breath, muscle and joint aches, and congested nose during the course of pregnancy. The second patient suffered from headache, nausea and vomiting, and diarrhea during pregnancy. Both patients denied infectious diseases during pregnancy and attributed symptoms to concomitant diseases and/or pregnancy problems.

### Concerns of COVID-19

Table 1 presents the results of the COVID-19 pandemic questionnaire answered by 94 obstetric patients at T1. The median of all questions was either 2.0 or 3.0 meaning “thoughts/a little concern” in the concern scale and the concern over time scale as well as “a little bit/moderately” in the impairment scale. Altogether, the median of the concern scale remained the same with 9.00 (8.00–12.00) at T1 and 9.00 (8.00–12.00) at T2 (p = 0.663). In contrast to that, the values of the concern over the time scale (p = 0.007), the impairment scale (p = 0.003) and the overall concern scale (sum of the concern scale, the concern over time scale and the impairment scale; p = 0.004; Wilcoxon test) increased significantly. The concern over time scale counted 8.00 (7.00–11.00) at T1 and 8.50 (7.00–11.00) at T2, the impairment scale 8.00 (7.00–11.00) at T1 and 9.00 (7.00–11.00) at T2, the overall concern scale 26.00 (22.00–32.00) at T1 and 28.00 (23.00–32.00) at T2. McDonald's Omega at T1 and T2 was 0.893/0.832 in the concern scale, 0.863/0.828 in the concern over time scale, 0.809/0.802 in the impairment scale and 0.846/0.803 of the overall concern scale (sum of the concern scale, the concern over time scale and the impairment scale).

The median of the Fear of COVID-19 scale (FCV-19S) at T2 was 12.00 (11.00–16.00) with a total score of 35 points representing maximum fear. McDonald’s Omega was 0.760. The results of the FCV-19S correlated significantly with the overall concern scale of the COVID-19 pandemic questionnaire at T1 (r_s_ = 0.283; p = 0.030) and the overall concern scale of the COVID-19 pandemic questionnaire at T2 (r_s_ = 0.405; p = 0.001). Furthermore, there were significantly positive correlations between FCV-19S and the following subscales of the COVID-19 pandemic questionnaire: Concern over time scale at T1 (r_s_ = 0.388; p = 0.002) and at T2 (r_s_ = 0.381; p = 0.003) and concern scale at T2 (r_s_ = 0.357; p = 0.005).

### Stress and coping inventory

The results of the different scales of the SCI were summarized in Table 3 representing rather low to low-medium stress levels and stress symptoms. Coping by believing in God or powers had a medium value and coping by drinking alcohol and/or smoking was scarcely present in our sample. The coping scales “positive coping”, “active coping” and “coping by support” had rather higher or high values with a significant increase of active coping from T1 to T2. McDonald's Omega at T1 and T2 was higher (in the most cases even clearly higher) than 0.627 in the different scales except for the coping by drinking alcohol and/or smoking scale at T1 with a McDonald's Omega of 0.542.

**Table 3:** Results of the stress and coping inventory at T1 and T2 in median and interquartile range

	T1		T2		
	Median	Interquartile range	Median	Interquartile range	P (Wilcoxon)
Stress caused by insecurity (total score: 49 points)	11.00	8.00–17.00	11.00	9.00–17.00	0.196
Stress caused by being overwhelmed (total score: 49 points)	11.00	9.00–17.00	12.00	10.00–17.00	0.972
Stress caused by loss (total score: 49 points)	7.00	7.00–10.00	7.00	7.00–10.00	0.663
Total load of stress (total score: 147 points)	30.50	24.00–42.00	32.00	26.00–43.00	0.615
Stress symptoms (total score: 52 points)	19.00	15.00–23.00	19.00	16.50–24.00	0.484
Positive Coping (total score: 16 points)	11.50	10.00–13.00	11.00	10.00–13.00	0.624
Active coping (total score: 16 points)	12.00	10.00–13.00	12.00	11.00–14.00	0.016
Coping by support (total score: 16 points)	15.00	14.00–16.00	15.00	13.00–16.00	0.388
Coping by believing in God or powers that be (total score: 16 points)	7.00	6.00–10.00	8.00	6.00–10.00	0.235
Coping by drinking alcohol and/or smoking (total score: 16 points)	4.00	4.00–6.50	4.00	4.00–6.00	0.311

p-values < 0.05 in the Wilcoxon test were considered as significant

The total load of stress of SCI at T1 correlated significantly with the overall concern scale of the COVID-19 pandemic questionnaire at T1 (r_s_ = 0.284; p = 0.008) as well as the subscales at T1 shown in Table 4.

**Table 4:** Correlation of subgroups of the stress and coping inventory at T1 with different subgroups of the COVID-19 pandemic questionnaire at T1

Spearman-Rho	Concern scale T1	Concern over time scale T1	Impairment scale T1	Sum score of the COVID-19 pandemic questionnaire T1
Stress caused by insecurity T1				
Correlation coefficient	.276^**^	.377^**^	.294^**^	.379^**^
p (two-sided)	0.009	0.000	0.005	0.000
N	89	89	89	87
Stress caused by being overwhelmed T1				
Correlation coefficient	.216^*^	.270^**^	.213^*^	.280^**^
p (two-sided)	0.038	0.009	0.042	0.008
N	92	92	92	90
Stress caused by loss T1				
Correlation coefficient	0.006	− 0.027	− 0.002	− 0.037
p (two-sided)	0.957	0.802	0.982	0.729
N	92	92	92	90
Total load of stress T1				
Correlation coefficient	.220^*^	.296^**^	.220^*^	.284^**^
p (two-sided)	0.038	0.005	0.038	0.008
N	89	89	89	87

^**^The correlation is significant at the 0.01 level (two-sided); *The correlation is significant at the 0.05 level (two-sided)

There was no significant correlation between the total load of stress measured by the SCI at T2 and the overall concern scale of the COVID-19 pandemic questionnaire at T2 (r_s_ = 0.047; p = 0.723). By comparison with FCV-19S, there were no significant correlations between stress or stress symptoms and FCV-19S. Only positive coping at T2 showed a significant negative correlation to FCV-19S (r_s_ = − 0.257, p = 0.046).

### Depression and anxiety

The median score of the EPDS measuring depression at T1 was 5.00 (2.00–7.00) with a insignificant small decrease at T2 (4.00 [2.00–7.00]; p = 0.312). McDonald’s Omega was 0.775 at T1 and 0.844 at T2. 10.64% (10 women) had an EPDS value of 10 or higher. The score of the EPDS at T1 correlated significantly with the overall concern scale of the COVID-19 pandemic questionnaire (r_s_ = 0.253; p = 0.02). This significant correlation was absent at T2 (r_s_ = 0.11; p = 0.41). There were also significant positive correlations between EPDS at T1 and the subscales concern over time at T1 (r_s_ = 0.212; p = 0.044) and impairment scale at T1 (r_s_ = 0.240; p = 0.022). The other subscales showed no correlation. Furthermore, there were no significant correlations of EPDS at T1 or T2 with FCV-19S. The median trait score of the ASI-3 was 11.50 (6.00–19.00; McDonald’s Omega 0.926) and indicated a rather low level of anxiety in view of an overall possible score of 72. The results of the COVID-19 pandemic questionnaire (concern scale, concern over time scale, impairment scale and overall concern scale) did not correlate significantly with the results of the ASI-3. In contrast, there was a significant correlation between the FCV-19S and the ASI-3 (r_s_ = 0.299, p = 0.026).

### Parental bonding and maternal self-confidence

The median of the PBQ was 6.00 (3.00–9.00; McDonald's omega 0.876) representing good maternal bonding. There were no significant correlations with the COVID-19 pandemic questionnaire and the FCV-19S. The median of the LMSCS was 119 (110–124; McDonald’s Omega 0.813) and showed a rather high self-confidence with a maximum of 144. There were no correlations to both COVID-19 questionnaires with one exception: The impairment scale of the COVID-19 pandemic questionnaire correlated negatively with the LMSCS (r_s_ = − 0.309, p = 0.016).

### Loneliness and isolation during social distancing

The LISD Scale hardly showed loneliness and isolation, as well as high social support in the trait and state scales. On all scales, the maximum score was 5.00. In detail, the mean score of state factor 1 (lonely and isolated) was 2.00 (1.67–2.67) and the mean state factor 2 score (connected and supported) was 4.67 (4.33–5.00). The mean trait factor 1 score (loneliness and isolation) was 1.50 (1.25–1.75), the mean trait factor 2 score (sociability and sense of belonging) was 4.20 (3.80–4.40), and the mean trait factor 3 score (social support and closeness) was 4.75 (4.50–5.00). McDonald's omega was 0.180 for the state factor 2 and 0.464 for trait factor 3. In the other scales, it was > 0.661. Table 5 presents possible correlations between the LISD Scale and the CPQ as well as the FVC-19S.

**Table 5:** Correlation of the results of the Loneliness and Isolation during Social Distancing Scale and the results of the COVID-19 pandemic questionnaire at T1 and T2 as well as the Fear of COVID-19-Scale at T2

Spearman-rho	Loneliness and Isolation during Social Distancing Scale				
	State factor 1 (lonely and isolated)	State factor 2 (connected and supported)	Trait factor 1 (loneliness and isolation)	Trait factor 2 (sociability and sense of belonging)	Trait factor 3 (social support and closeness)
Concern scale T1					
Correlation coefficient	0.016	0.034	0.143	− 0.037	0.176
p (two-sided)	0.905	0.801	0.278	0.782	0.182
N	60	59	59	59	59
Concern over time scale T1					
Correlation coefficient	0.076	0.077	0.171	− 0.043	0.121
p (two-sided)	0.562	0.56	0.196	0.745	0.363
N	60	59	59	59	59
Impairment scale T1					
Correlation coefficient	.373**	− .282*	.382**	− 0.091	− 0.095
p (two-sided)	0.003	0.03	0.003	0.491	0.476
N	60	59	59	59	59
Overall concern scale T1					
Correlation coefficient	0.234	− 0.057	.296*	− 0.122	0.124
p (two-sided)	0.078	0.676	0.025	0.367	0.358
N	58	57	57	57	57
Concern scale T2					
Correlation coefficient	0.098	− 0.027	0.121	− 0.128	0.016
p (two-sided)	0.454	0.838	0.357	0.331	0.906
N	60	59	60	60	60
Concern over time scale T2					
Correlation coefficient	0.157	0.01	0.117	− 0.051	0.194
p (two-sided)	0.236	0.939	0.379	0.701	0.141
N	59	58	59	59	59
Impairment scale T2					
Correlation coefficient	.507**	− .285*	.355**	0.025	− 0.094
p (two-sided)	0	0.027	0.005	0.848	0.473
N	61	60	60	60	60
Overall concern scale T2					
Correlation coefficient	.332*	− 0.119	.294*	− 0.121	0.082
p (two-sided)	0.01	0.373	0.024	0.361	0.538
N	59	58	59	59	59
Fear of COVID-19 questionnaire					
Correlation coefficient	0.154	0.065	0.192	− 0.088	0.093
p (two-sided)	0.236	0.621	0.142	0.502	0.478
N	61	60	60	60	60

^**^The correlation is significant at the 0.01 level (two-sided); *The correlation is significant at the 0.05 level (two-sided)

### Biomarker

Table 6 shows the correlation of stress hormones (cortisol, adrenaline, norepinephrine, dopamine) as well as the infection parameter IL-6 with the results of the questionnaires at T1. We found a negative correlation between the dopamine level in the periperial blood and the total load of stress (r_s_ = − 0.288, p = 0.007), stress symptoms (r_s_ = − 0.231, p = 0.032) as well as the overall concern scale in the COVID-19 pandemic questionnaire (r_s_ = − 0.212, p = 0.047). Further, the levels of IL-6 correlated negatively with the total load of stress (r_s_ = − 0.227, p = 0.034).

**Table 6:** Correlation of various biomarkers with the results of the different questionnaires (Stress and coping inventory [SCI], COVID-19 pandemic questionnaire [CPQ] and Edinburgh Postnatal Depression Scale [EPDS]) at T1

Spearman-Rho	Cortisol	Adrenaline	Norepi-nephrine	Dopamine	IL-6
SCI					
Stress caused by insecurity					
Correlation coefficient	0.014	0.056	0.146	− .302**	− .215*
p (two-sided)	0.9	0.61	0.18	0.004	0.045
N	87	86	86	87	87
Stress caused by being overwhelmed					
Correlation coefficient	0.104	− 0.096	0.047	− .260*	− 0.204
p (two-sided)	0.33	0.371	0.663	0.013	0.054
N	90	89	89	90	90
Stress caused by loss					
Correlation coefficient	0.1	− 0.168	− 0.009	− 0.033	− 0.159
p (two-sided)	0.348	0.115	0.935	0.759	0.133
N	90	89	89	90	90
Total load of stress					
Correlation coefficient	0.098	− 0.083	0.085	− .288**	− .227*
p (two-sided)	0.366	0.448	0.439	0.007	0.034
N	87	86	86	87	87
Stress symptoms					
Correlation coefficient	0.027	0.049	0.022	− .231*	− 0.202
p (two-sided)	0.803	0.654	0.845	0.032	0.063
N	86	85	85	86	86
Concern scale					
Correlation coefficient	0.094	0.025	− 0.065	− 0.133	− 0.199
p (two-sided)	0.379	0.817	0.548	0.212	0.061
N	90	89	89	90	90
CPQ					
Concern over time scale					
Correlation coefficient	0.102	0.111	0.054	− .257*	− 0.167
p (two-sided)	0.337	0.299	0.612	0.015	0.115
N	90	89	89	90	90
Impairment scale					
Correlation coefficient	0.027	0.001	0.018	− 0.102	− 0.097
p (two-sided)	0.8	0.993	0.87	0.337	0.363
N	90	89	89	90	90
Overall concern scale					
Correlation coefficient	0.108	0.088	0.017	− .212*	− 0.196
p (two-sided)	0.315	0.416	0.875	0.047	0.068
N	88	87	87	88	88
EPDS					
Correlation coefficient	− 0.021	0.047	0.028	− .232*	− 0.046
p (two-sided)	0.844	0.665	0.795	0.029	0.669
N	89	88	88	89	89

^**^The correlation is significant at the 0.01 level (two-sided); *The correlation is significant at the 0.05 level (two-sided)

## Discussion

Although the extension of the COVID-19 pandemic is worldwide, the health systems as well as the regulations and restrictions for pandemic control (school and workplace closures, cancellation of public events and gatherings, stay at home restrictions, face coverings, international and domestic travel, testing and contact tracing, public information campaigns, vaccination policy and income support and dept relief) are different in each country [3–5]. The University Hospital in Würzburg is one of the larger University Hospitals in Germany and is therefore representative of the German population. In our study, the scores of the COVID-19 pandemic questionnaire showed overall a low to moderate overall level of concern, whereas the level of concern increased over the course of the pandemic, i.e., between delivery stay (T1) and 3 to 6 months postpartum (T2). This corresponds to findings in other studies [60], although most of studies show results from one date and no comparison of stress level to a later date during the ongoing pandemic [60–64].

The SCI results showed low stress levels, while a significant increase in active coping mechanisms could be detected between delivery stay to postpartum assessment (from T1 to T2), especially in the following subgroups: women under 35 years, academics (university graduates), body mass index (BMI) < 25 kg/m^2^, caesarian section, first birth and high-risk pregnancies. Coping strategies are known to be an important factor for maternal mental health [65–67].

At the delivery stay (T1), higher total load of stress related to more overall COVID-19-related concern, especially stress by insecurity and stress by being overwhelmed. This effect dissolves over time and cannot be detected anymore at the postpartum assessment (T2), which might be interpreted as an effect of habituation and of the improvement of coping strategies during the pandemic as described above [68–70].

No significant correlations between psychosocial stress or stress symptoms of SCI and FCV-19S could be shown, considering the fact, that the FCV-19S focusses on general fear concerning COVID-19 and not specific worries of pregnant women during the pandemic as the CPQ [43]. A possible reason to explain the lack of relationships could be that the stressors for our group are the specific concerns relating to pregnancy and the obstetric situation and not a generally raised level of fear. In contrast to our observed low score of the FCV-19S, another study reported high levels [71]. This difference could be due to differences in ethnic and socio-demographic aspects in the tested population. It is also possible that the time period studied during the COVID-19 pandemic, the week of pregnancy and the care capacity of the health care system are important confounding factors.

A large trial with more than 600 women in the UK after birth resulted in elevated levels of depression and anxiety during the COVID-19 pandemic [72] which is supported by other studies [41, 73–78].

In contrast, in our study we have found relatively low scores of the EPDS and the ASI-3. The rate of about 10% estimated depression detected by the EPDS was in the normal prevalence range (about 7–13%) in pregnancy and postpartum [9, 79]. At first this may seem to contradict the previously mentioned studies [41, 72–78]. However, due to our study design (Fig. 1) we were unable to compare to pre-pandemic values. Instead, we investigated relationships of depression and COVID-19 specific factors. More COVID-19 related concerns related to higher depression, but not anxiety scores. In contrast, COVID-19 related fear correlated positively to anxiety, but not depression. There seems to be a difference between obstetric concerns in the face of COVID-19 pandemic and general fear of COVID-19, although they correlated to some degree.

In the face of the SARS-CoV-1 outbreak, Lee and colleagues have shown that anxiety and depression did not increase compared to pre-outbreak levels. The authors attribute this to increased social support [80]. This might also be the underlying reason for this study sample´s low scores in anxiety and depression. Social support during pregnancy does not only have an influence on mental health symptoms such as depression but also on pregnancy outcome [81]. Although some studies report low social support and the consequent negative impact on women in late pregnancy and postpartum during the COVID-19 pandemic [82, 83], pregnant women may also have benefited from increased flexibility of work schedules and jobs due to COVID-19 pandemic restrictions, such as themselves or their husbands' ability to work from home. Strengthening this line of evidence, our results show low to low-medium loneliness and social isolation and high social support on both the state and the trait level. Being lonely and socially isolated (state and trait) was associated with higher impairment scores both during the delivery stay and several months postpartum, while being socially connected and supported (state) related to lower impairment. Note that interpretation of the indicators of social support are limited by a low reliability. This may have been caused by extremely skewed score distributions and high scores for being connected and supported (state) and for social support and connectedness (trait 3). Nonetheless, these findings emphasize the effect of social support and particularly isolation and loneliness on the level of mental health impairment.

Although many factors influence the mother-infant-bonding with stress levels caused by the COVID-19 pandemic potentially being among them [84, 85], we detected a good maternal-infant bonding in our study, independent of concerns and fears regarding SARS-CoV-2 and the COVID-19 pandemic. This also corresponds well with the observed low stress levels. However, the impairment by the COVID-19 pandemic correlated negatively with maternal self-confidence. This was also the finding of Vazquez-Vazquez and co-authors. They suggest that maternal lack of contact with other mothers through the restrictions caused by the COVID-19 pandemic had an impact on the assessment of maternal self-efficacy [86].

Here, we found a significant inverse relationship of peripheral dopamine level with level of stress and level of concern in the COVID-19 pandemic questionnaire. There were also similar results for the correlation between peripheral dopamine levels and depression scores. These findings support the hypothesis that dopamine plays an important role in modulating stress-coping mechanisms although most of studies focus on dopamine levels in the central nervous system and not peripheral levels [87–91].

Our study showed a correlation of IL-6 and elevated stress levels, which emphasize the findings of other authors, that higher levels of IL-6 are associated with mental health problems such as depression and anxiety [92–94].

For serum levels of cortisol, adrenaline and noradrenaline we did not find any significant correlation. The results of other studies are heterogeneous, levels of these hormones are shown to be strongly dependent on individual baselines and circadian rhythm as well as the time interval to stressors and intensity of stressors, which our study did not take into account [95, 96].

## Strength, limitations and future directions

Our study included patients at the beginning of the pandemic and collected data of the postpartum period what allows us to observe changes during the course of the pandemic. Another strength is that we also considered the maternal self-efficacy and parental bonding as well as biomarkers. A limitation of our study was that there were no clearly defined survey time or conditions for the survey as well as the maternal blood sample, which owed to the course of labour and the workflow in delivery rooms. This may have lead to a blur in the obtained data, however we rate this to be of minor influence on our overall finding. Furthermore, the comparability of studies focusing on impairment of mental health of pregnant and postpartum women due to the COVID-19 pandemic may be limited since there are different tools to measure anxiety in pregnancy [97–99]. Our study design offers the opportunity to do a reassessement of the included women as well as an investigation of a new cohort of women for example after specific changes during the pandemic such as the implementation and availability to vaccination.

## Conclusion

In our study we found increased levels of concern of obstetric patients regarding the COVID-19 pandemic during the course of the pandemic in Germany that correlates particularly with stress and depression levels. Our results suggest that raised levels of stress are rather of situational nature and not a result of generally raised levels of fear and depression. The women of our study population experienced a raised level of active coping over time as well as good levels of parental bonding, neither being adversely affected by measured impairment of the pandemic. In contrast, maternal self-efficacy was influenced in part by the restrictions imposed by the pandemic.

Our findings support previous work studying the psychological effects of the pandemic and an increased risk for mental health problems. They emphasize the special situation of pregnant women during this period and as well the need for monitoring, prevention and intervention.

## Data Availability

The datasets generated and/or analysed during the current study are available from the corresponding author on reasonable request.

## Abbreviations

ASI-3: Anxiety Sensitivity Index-3
BMI: Body mass index
COVID-19: Coronavirus induced disease 2019
CPQ: COVID-19 pandemic questionnaire
ELISA: Enzyme-linked immunosorbent assays
EPDS: Edinburgh Postnatal Depression Scale
FCV-19S: Fear of COVID-19 Scale
IL-6: Interleukin-6
LISD: Loneliness and Isolation during Social Distancing Scale
LMSCS: Lips maternal self-confidence scale
MERS: Middle East respiratory syndrome
MERS-CoV: Middle East respiratory syndrome coronavirus
PBQ: Parental Bonding Questionnaire
RT-PCR: Reverse transcriptase polymerase chain reaction
RT-qPCR: Reverse transcriptase quantitative polymerase chain reaction
SARS: Severe acute respiratory syndrome
SARS-CoV: Severe acute respiratory syndrome coronavirus
SARS-CoV-2: Severe acute respiratory syndrome coronavirus 2
SCI: Stress and coping inventory
SD: Standard deviation
WHO: World Health Organization
